# Influence of Surfactant and Lipid Type on the Physicochemical Properties and Biocompatibility of Solid Lipid Nanoparticles

**DOI:** 10.3390/ijerph110808581

**Published:** 2014-08-20

**Authors:** Carine Dal Pizzol, Fabíola Branco Filippin-Monteiro, Jelver Alexander Sierra Restrepo, Frederico Pittella, Adny Henrique Silva, Paula Alves de Souza, Angela Machado de Campos, Tânia Beatriz Creczynski-Pasa

**Affiliations:** 1 Departamento de Ciências Farmacêuticas, Programa de Pós-Graduação em Farmácia, Universidade Federal de Santa Catarina, Florianópolis, SC 88040-900, Brazil; E-Mails: carine@dalpizzol.com (C.D.P.); filippinmonteiro@gmail.com (F.B.F.-M.); adnyh@yahoo.com.br (A.H.S.); paula.as@hotmail.com (P.A.S.); angelacampos@ufsc.br (A.M.C.); 2 Progama de Pós-Graduação em Engenharia de Materiais, Universidade Federal de Santa Catarina, Florianópolis, SC 88040-900, Brazil; E-Mail: jelver.sierra@gmail.com; 3 Departamento de Análises Clínicas, Universidade Federal de Juiz de Fora, Juiz de Fora, MG 36036-900, Brazil; E-Mail: fredpittella@gmail.com

**Keywords:** solid lipid nanoparticles, cytotoxicity, surfactant, lipid, biocompatibility

## Abstract

Nine types of solid lipid nanoparticle (SLN) formulations were produced using tripalmitin (TPM), glyceryl monostearate (GM) or stearic acid (SA), stabilized with lecithin S75 and polysorbate 80. Formulations were prepared presenting PI values within 0.25 to 0.30, and the physicochemical properties, stability upon storage and biocompatibility were evaluated. The average particle size ranged from 116 to 306 nm, with a negative surface charge around −11 mV. SLN presented good stability up to 60 days. The SLN manufactured using SA could not be measured by DLS due to the reflective feature of this formulation. However, TEM images revealed that SA nanoparticles presented square/rod shapes with an approximate size of 100 nm. Regarding biocompatibility aspects, SA nanoparticles showed toxicity in fibroblasts, causing cell death, and produced high hemolytic rates, indicating toxicity to red blood cells. This finding might be related to lipid type, as well as, the shape of the nanoparticles. No morphological alterations and hemolytic effects were observed in cells incubated with SLN containing TPM and GM. The SLN containing TPM and GM showed long-term stability, suggesting good shelf-life. The results indicate high toxicity of SLN prepared with SA, and strongly suggest that the components of the formulation should be analyzed in combination rather than separately to avoid misinterpretation of the results.

## 1. Introduction

The development of nanoparticles has received considerable attention in the pharmaceutical sciences field due to the potential to modulate the pharmacological effect of nanoencapsulating active substances [1,2,3]. Lipid nanoparticles, in particular, represent promising systems for enhanced incorporation of hydrophobic compounds into the lipid matrix. Among these nanostructures, solid lipid nanoparticles (SLNs) are characterized for presenting a solid lipid matrix at room temperature, and have been extensively used in pharmaceutical and cosmetic formulations [4,5,6]. In addition, the inexpensive and facile method of preparation allows the combination of various components towards the formation of SLNs [7,8].

Usually, the preparation of SLNs includes the homogenization of a lipid phase and an aqueous phase at high temperatures for the production of a fine-disperse oil-in-water emulsion [8,9]. The SLNs are obtained by cooling the emulsion below the crystallization temperature of the main lipid, resulting in the formation of a solid lipid matrix [8,9]. In fact, the choice of the lipid and excipients in a SLN formulation might be critical for the efficient preparation and application of these nanoparticles. The lipids normally employed for the production of SLN include triglycerides (e.g., tripalmitin), partial glycosides (e.g., glyceryl monostearate), fatty acids (e.g., stearic acid), sterols (e.g., cholesterol) and waxes (e.g., cetyl palmitate) [10,11]. Besides the matrix lipid, the surfactants are used for further stabilization of the formulation and comprise poloxamers, lecithins, polysorbates and polyethoxylated monoglycerides [8]. Both physiological lipids and surfactants are usually well accepted [8,10]. However, there have only been a few studies concerning the biological/toxicological aspects of these components in combination, as drug-free nanocarriers [12].

Although several reports suggest negligible cytotoxicity arising from the use of SLNs [13,14,15,16], the composition of the nanoparticles assessed in these experiments varies in nature, percentage and method of preparation. In contrast, *in vitro* cytotoxicity in fibroblasts, macrophages and keratinocytes was found with the application of SLN composed of stearic acid or hard lipids as core material and various surfactants [17]. It was shown that the cell viability was greatly affected by the nature of the lipid and the concentration of surfactant. Accordingly, our previous work reported a high toxicity of SLNs prepared with sodium dodecyl sulfate as surfactant, suggesting the dependence of the cytotoxicity on the characteristics of the excipients (lipids and surfactants) [18]. Moreover, the physical state of the matrix lipid of SLN may also disturb the cell viability in addition to the influence of the size and shape of nanoparticles in the final biological effect [12].

In this study, great importance was attached to the evaluation of biocompatibility and toxicity of SLN components in combination as plain nanoparticles and preformulation constituents. In addition, the elucidation of cell responses to the SLN application may provide valuable information for the development of safe and efficient formulations. To this end, we prepared SLNs composed of three types of major matrix lipids (tripalmitin, glyceryl monostearate or stearic acid), stabilized with two surfactants (lecithin S75 and polysorbate 80) in three different concentrations. This set of SLNs was evaluated in terms of physicochemical properties, stability upon storage and biocompatibility, which included extensive analysis of the mechanisms whereby the SLNs interfere with the cell cycle, mitochondrial functioning, enzymes related to the regulation of cell death as well as erythrocyte hemolysis.

## 2. Experimental Section

### 2.1. Materials and Cell Line

Dimethyl sulfoxide (DMSO) and polysorbate 80 were purchased from Synth (Diadema, SP, Brazil). Lecithin S75 and ethanol were provided by Lipoid (Ribeirão Preto, SP, Brazil) and Lafan (São Paulo, SP, Brazil), respectively. Tripalmitin was kindly provided by Sasol (São Paulo, SP, Brazil), while stearic acid and glyceryl monostearate were purchased from Galena (Campinas, SP, Brazil). Fetal bovine serum (FBS), penicillin, streptomycin, Dulbecco’s modified Eagle’s medium (DMEM) and RPMI 1640 were purchased from Invitrogen (Carlsbad, CA, USA). (3-(4,5-Dimethylthiazol-2-yl)-2,5-diphenyltetrazolium bromide (MTT), acridine orange, propidium iodide, ethidium bromide, sodium bicarbonate, and (4-(2-hydroxyethyl)-1-piperazineethanesulfonic acid) (HEPES) were supplied by Sigma Chemical Co. (St. Louis, MO, USA). A murine fibroblast cell line (NIH/3T3—ATCC CRL-1658) was purchased from Banco de Células do Rio de Janeiro (Rio de Janeiro, RJ, Brazil).

### 2.2. Solid Lipid Nanoparticles (SLN) Preparation

The formulations were prepared using the ultrasound method as previously described by Patti [19]. Briefly, the matrix lipid (glycerol monostearate or tripalmitin or stearic acid (2% (w/v)) and lecithin S75 surfactant (0.1%–0.3%) were heated for about 56–70 °C. Next, the PBS buffer aqueous solution containing polysorbate 80 (0.4%–1.2% (w/v)), previously heated at the same temperature of the lipid phase, was mixed in the oil phase under stirring. After that, the sonication probe (6 mm diameter) of an ultrasonic processor (Vibracells, Newtown, CT, USA) was placed in the pre-emulsion and set to produce an output power with 70% amplitude for 3 min at 4 °C, leading to droplet breakage by acoustic cavitation and subsequent nanoparticles formation.

### 2.3. Dynamic light scattering and zeta potential

The particle size, polydispersity index (PI) and zeta potential of SLN were measured using a Zetasizer Nano ZS with back scattering detector (Malvern Instruments, Worcestershire, UK), equipped with 173° scattering angle. The measurements were taken at 25 °C, and the data obtained from the rate of decay in the photon correlation function were analyzed with a cumulant method to obtain the corresponding hydrodynamic diameters and polydispersity indices (PI) (μ/G^2^) of the SLN. The zeta potential values were calculated from the mean of electrophoretic mobility values using Smoluchowski’s equation. DLS measurements were used to evaluate the stability of nanoparticles under storage conditions (4 °C and 25 °C) during 60 days.

### 2.4. Transmission Electron Microscopy (TEM)

SLN dispersions were diluted with purified water (1:10) prior to the observation using TEM. The samples were set on a copper metal substrate coated with carbon (CF200-Cu, 300 square mesh cupper, EMS, Hatfield, PA, USA). The material was dried with nitrogen flow and left at room temperature for 12 h. The particles were visualized at 100,000 times magnification in a JEM-1011 TEM model (JEOL, Peabody, MA, USA).

### 2.5. Cell Culture

Murine fibroblast cell line (NIH/3T3) were cultured in DMEM supplemented with 10% heat-inactivated fetal bovine serum, 100 U·mL^−1^ penicillin, 100 μg·mL^−1^ streptomycin, 10mM HEPES and maintained at 37 °C in a 5% CO_2_ humidified atmosphere and pH 7.4. Every 2–3 days, cells were passaged by removing 90% of the supernatant, which was replaced by fresh medium. In all experiments, viable cells were checked at the beginning of the experiment by Trypan Blue exclusion.

### 2.6. Cell Viability Assay

The cell viability study was assessed performing the (4,5-dimethylthiazol-2-yl)-2,5-diphenyl-tetrazolium bromide (MTT) assay [11]. This test evaluates the mitochondrial function as a measurement of cell viability, which permits the detection of cells before they lose their integrity and shape. Briefly, NIH/3T3 cells (1 × 10^4^ mL^−1^) were seeded in 96-well plate and incubated for 24 h. Next, the culture medium was replaced by a fresh one with SLN in a concentration range of 50 to 1000 µg·mL^−1^. After 24 h incubation, cells were washed with fresh culture medium and 5 mg·mL^−1^ of MTT were added followed by incubation for 2 h at 37 °C. The precipitated formazan was dissolved in DMSO and the absorbance was measured at 540 nm using a micro-well system reader (Organon Teknika, Turnhout, Belgium). The cytotoxic concentration (CC_50_) values were calculated using a Hill concentration-response curve.

### 2.7. Cell Cycle Analysis

The cell cycle of treated cells was analyzed by flow cytometry Yang *et al.* [20]. Briefly, cells (1 × 10^6^/well) were seeded in a 12-well plate for 24 h. The medium was then replaced and the SLN were added at the cytotoxic concentration (CC_50_) for a further 24 h incubation. Next, cells were centrifuged at 400× g for 10 min at room temperature. After that, the cells were washed with PBS and centrifuged under the same conditions as before. The supernatant was removed and cells were fixed with 70% ethanol for 30 min at 4 °C. Following that, PBS with 2% of BSA was added and centrifuged for 10 min at 400× g. The supernatants were removed and cells were permeabilized with lysis buffer (0.1% Triton-X in PBS) and 0.5 μL of RNase (100 μg/mL). DNA content stained with propidium iodide (20 μg/mL) was analyzed using the FACS Canto flow cytometry equipment (Becton Dickinson, San Jose, CA, EUA). The cell population in each phase of the cell cycle was determined using WinMDI 2.9 software.

### 2.8. Morphological Identification for Cell Death

The identification of apoptosis or necrosis was carried out by a double staining with acridine orange (3,6-dimethylaminoacridine) and ethidium bromide (3,8-diamino-5-ethyl-6-phenylphenanthridinium bromide) method [21]. For that, 1 × 10^6^ cells/well were seeded in 12-well plates and incubated with CC_50_ concentrations of each formulation for 24 h. Control group was incubated only with growth medium. Medium was then removed and cells were washed with PBS, followed by the double staining with acridine orange and ethidium bromide and visualization with fluorescence microscopic imaging (Eclipse TS100, Nikon, Melville, NY, EUA). Images were obtained at 200 × magnification and analyzed by Image J software using a cell counter plugin (National Institute of Health, Bethesda, MD, USA) for evaluation of morphological alterations and identification of cell death type. Accordingly, (a) viable cells appeared to have green nucleus with intact structure; (b) cells with bright-green nucleus and chromatin condensation (early apoptosis), in addition to dense orange areas of chromatin condensation (late apoptosis) were considered apoptosis; and (c) cells presenting orange intact nucleus depicted secondary necrosis.

### 2.9. Erythrocyte Hemolysis

Blood samples were obtained from healthy donors (25–32 years old) by venipuncture and collected into test tubes containing 124 mM sodium citrate (9:1 (v/v), blood:sodium citrate). The erythrocytes were immediately separated by centrifugation at 2000× g for 5 min and washed three times with four volumes of PBS. Erythrocytes collected from the blood were re-suspended in normal saline solution. 1% (w/v) dispersions of SLN were immediately mixed with saline and incubated with a sample of the erythrocyte suspension at 37 °C with gentle tumbling of the test tubes. After 1 h of incubation, samples were centrifuged for 5 min at 2000× g. The supernatant was then collected and mixed with dichloromethane to separate the SLN in the organic phase. The aqueous phase absorbance was measured at 415 nm to determine the percentage of hemolyzed cells. Hemolysis was induced with double-distilled water and taken as positive control [1].

### 2.10. Statistical Analysis

The results were presented as means ± standard error of the mean (SEM) of triplicates from at least three independent experiments. Statistical analysis of differences among the formulations and treatments were performed using ANOVA, followed by Dunnett’s test in which more than two groups were compared to each other and a *p* value less than 0.05 was considered significant.

## 3. Results and Discussion

### 3.1. Solid Lipid Nanoparticles (SLN) Preparation and Characterization

Solid lipid nanoparticle dispersions were successfully prepared using the ultrasound method. The composition of each formulation of plain SLN is presented in Table 1 and identified as Formulations F1–F9. For this study, three types of major matrix lipids (tripalmitin, glyceryl monostearate or stearic acid) were chosen along with two main surfactants (lecithin S75 and polysorbate 80) commonly employed for SLN preparation. To investigate the influence of the surfactant amount in each formulation, the concentration of polysorbate 80 varied from 0.4% to 1.2% (w/v), while lecithin S75 varied from 0.1% to 0.3% (w/v).

**Table 1:** Composition of SLNs.

SLN *	Tripalmitin (%)	Glycerol Monostearate (%)	Stearic Acid (%)	Polysorbate 80 (%)	Lecithin S75 (%)
F1	2	-	-	0.4	0.1
F2	2	-	-	0.8	0.2
F3	2	-	-	1.2	0.3
F4	-	2	-	0.4	0.1
F5	-	2	-	0.8	0.2
F6	-	2	-	1.2	0.3
F7	-	-	2	0.4	0.1
F8	-	-	2	0.8	0.2
F9	-	-	2	1.2	0.3

* % of final volume in PBS buffer.

All the formulations were submitted to characterization by dynamic light scattering and transmission electron microscopy. The z-average size, polydispersity index (PI) and zeta potential of the formulations F1 to F6 are listed in Table 2. Interestingly, the formulations containing stearic acid (F7 to F9) could not be measured by dynamic light scattering (DLS) due to the reflective feature of these SLN impairing the correct measurement.

**Table 2:** Characteristics of SLNs: particle size, PI and zeta potential.

SLN	z-Average Diameter (nm) *	PI *	Zeta Potential (mV) *
F1	306 ± 9.9	0.26 ± 0.06	−15 ± 0.7
F2	167 ± 5.3	0.27 ± 0.02	−14 ± 1.4
F3	116 ± 6.9	0.25 ± 0.02	−12 ± 0.7
F4	232 ± 13	0.26 ± 0.03	−13 ± 1.8
F5	148 ± 9.3	0.26 ± 0.02	−12 ± 1.3
F6	135 ± 7.5	0.30 ± 0.07	−11 ± 0.8

* (mean ± SD, *n* = 3).

The SLN dispersions presented PI values within 0.25 to 0.30, while the z-averaged size ranged from 116 to 306 nm. The size of the particles was found to be inversely correlated to the surfactant concentration, a trend that was independent of the matrix lipid type. Conversely, the increase in the surfactant concentration did not promote a significant trend to the zeta potential of the SLNs (*p* < 0.05), which presented negative surface charge with values of −15 to −10 mV.

**Figure 1:**
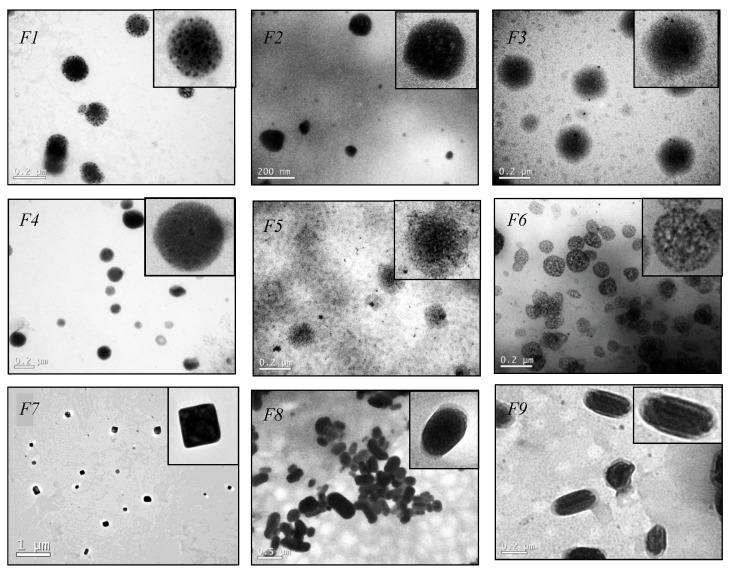
Representative transmission electron microscopic (TEM) images of F1–F9.

In addition to DLS measurements, TEM analysis was performed to obtain details about the morphology of the nanoparticles. As shown in Figure 1, TEM images revealed SLNs with spherical morphology for formulations F1 to F6. The nanoparticles of the F5 and F6 appear to be porous that might be related with the high surfactant concentration containing in these formulations. Further studies are necessary to understand this effect.

Interestingly, formulations F7 to F9 presented squared/rod shapes. This particular shape can be explained by solute-solvent interactions [22]. The crystal modification can be affected in nanoparticles preparation when the lipid is heated followed by cooling. Addition of surfactant can force the stearic acid to crystallize regardless of the crystallization conditions and the nature of the solvent [22]. A cubic phase occurs in some systems with chain lengths above C14, which structure consist of two interpenetrating networks of rod-like aggregates and it has been suggested from theoretical point of view that there are two fundamentally different alternatives for cubic lipid-water structures, (i) structures with continuous regions of both water and hydrocarbon chains and (ii) structures composed of closed aggregates of “oil-in-water” or “water-in-oil’’ type, this added to the effects of surfactant could explain the formation of particles with regions showing domains with different electron-densities seen on TEM, however deeper studies should be performed to understand the formation of the presented SLNs [23,24].

### 3.2. Stability of SLN upon Storage

**Figure 2:**
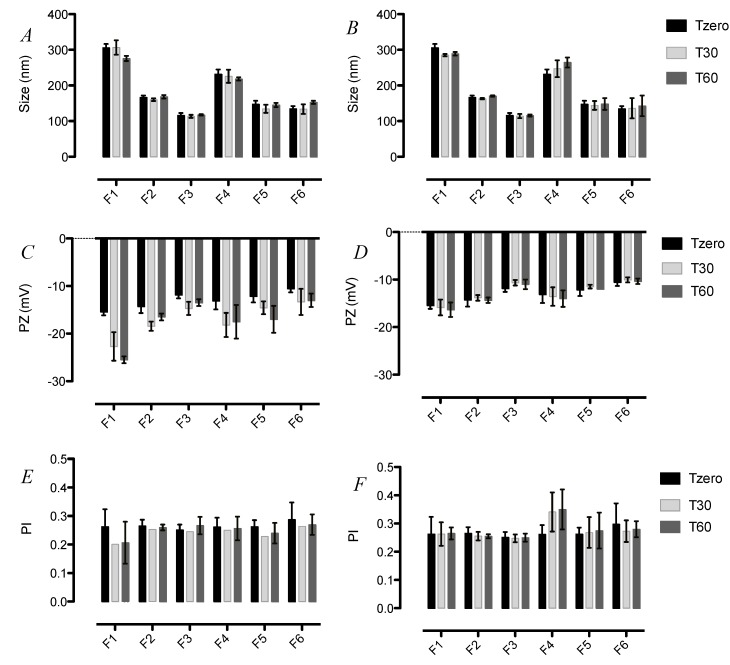
Stability of SLN upon storage. (**A**) Size of SLN F1-F6 over 60 days at 25 °C; (**B**) Size of F1-F6 SLN over 60 days at 4 °C; (**C**) Zeta potential of SLN F1-F6 over 60 days at 25 °C; (**D**) Zeta potential of SLN F1-F6 over 60 days at 4 °C; (**E**) PI of SLN F1-F6 over 60 days at 25 °C; (**F**) PI of F1-F6 SLN over 60 days at 4 °C.

The information regarding the stability of SLN formulations upon storage is important for their quality control for pharmaceutical application. Thus, the size, PI and zeta potential of the formulations F1–F6 were monitored over time by DLS following storage at 4 and 25 °C (Figure 2). Neither the size nor the PI of all tested formulations showed significant changes for at least 60 days at both 4 and 25 °C. The good colloidal stability observed for these formulations might be related to the protection by the surfactant used, *i.e.*, polysorbate 80 on the particle surface is known to provide excellent steric hindrance, which prevents particle aggregation [9,25].

On the contrary, none of the formulations showed the increase pattern at 4 °C as presented. In addition, the measurements of zeta potential revealed a significant increase in the surface charge of the formulations F1 and F5 stored for 60 days at 25 °C (Figure 2C). These results were also observed by other groups [26,27]. Such instability may be due to external parameters such as light and temperature, which can change the crystalline structure of lipids followed by changes in zeta potential and size, being of great importance in the maintenance of long-term stability [26] in Figure 2D.

### 3.3. Cell Viability Assay

Understanding the effect of different components combined as nanoparticles on the viability and cellular pathways may provide useful information for the choice of a final formulation. We therefore performed extensive analysis of the formulations on fibroblast cells viability and searched for further mechanisms of any eventual cytotoxicity caused by SLN formulations.

The cytotoxicity profile of SLNs in fibroblasts was investigated by the MTT assay under *in vitro* conditions. The viability of NIH/3T3 cells was determined after incubation of cells for 24 h with SLNs, and the CC_50_ values are shown in Table 3. Due to the fact that surfactants are generally regarded as potentially irritant or poorly tolerated [27], the concentration of these excipients varied among the formulations. When tripalmitin was used as the matrix lipid (F1–F3), there was a significant tendency for cell viability to reduce with the increase of surfactant concentration. Although the correlation is positive for the size reduction for the formulations F1–F3, the SLN F4–F9 did not present this size trend.

**Table 3:** CC_50_ of SLN in fibroblast cells NIH/3T3.

SLN	CC_50_ (μg·mL^−1^) *
F1	1420 ± 20
F2	730 ± 12
F3	602 ± 39
F4	410 ± 27
F5	480 ± 32
F6	260 ± 15
F7	330 ± 19
F8	210 ± 38
F9	310 ± 25

* CC_50_ in μg·mL^−1^ of total lipids. Data are expressed as percent of control (mean ± SE, *n* = 3).

This indicates that the combination of components should be considered as the primary parameter for cytotoxicity profiles instead of the size. It is also important to note that previous studies [17,28,29,30,31,32] have indicated negligible *in vitro* and *in vivo* toxicity of SLNs prepared with commonly used lipids and surfactants such as the SLNs prepared for this study. The contrasting results presented here strongly suggest that the analysis of components should be considered in combination rather than separately.

An additional important finding is related to the morphology of the formulations F7 to F9, which nanoparticles have squared-rod shape. This geometric shape might influence the cellular uptake increasing the cytotoxicity, as already observed by other authors [33,34,35].

### 3.4. Cell Cycle Analysis

**Figure 3:**
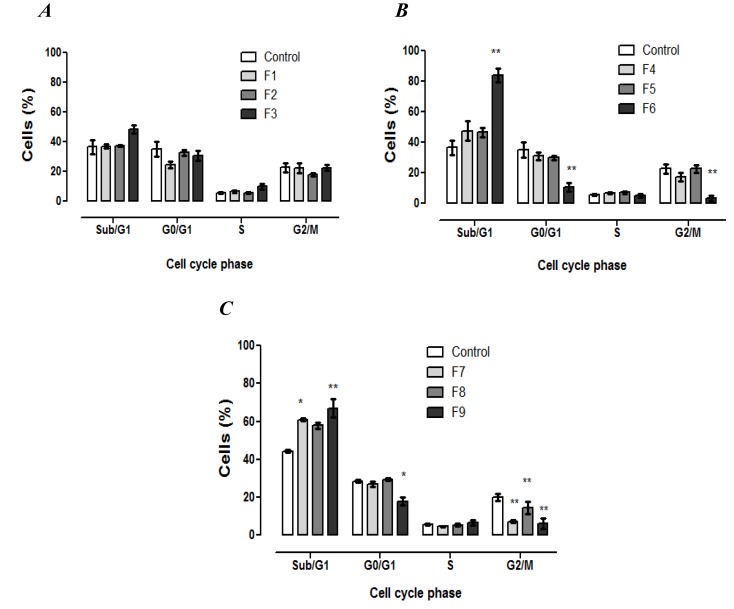
Fibroblasts cell cycle analysis after 24 h of exposure to formulations. The percentage of cells in each phase of the cell cycle stained with PI solution (2 μg·mL^−1^) and analyzed by flow cytometry. (**A**) Formulations containing tripalmitin as matrix lipid; (**B**) formulations containing glycerol monostearate as matrix lipid; and (**C**) formulations containing stearic acid as matrix lipid. Data are expressed as mean ± SE of 3 independent experiments. ANOVA followed by Dunnett’s test (* *p* < 0.01, ** *p* < 0.001 against control).

The effect of SLN formulations on cell cycle distribution of fibroblasts was investigated by flow cytometry in order to determine if the cytotoxicity was associated with alterations in the progression of cell cycle. Propidium iodide (PI) is capable of binding and labeling the DNA for the evaluation of the content and damage of cellular DNA. With the DNA labeled the identification of hypodiploid cells is feasible. As shown in Figure 3, 24 h after the treatment with SLNs F6–F9, a significant decrease in the cells on the G2/M phase was observed.

Consequently, there was an increase in subdiploid DNA content related to the Sub-G1 phase for the cells treated with F6, F7 and F9, which was statistically significant compared to the control group. On the other hand, the tested SLNs did not induce the cell cycle arrest. Overall, the results indicate that the formulations presenting the CC_50_ value lower than 350 μg·mL^−1^ induced DNA fragmentation as a consequence of the increase in Sub-G1 phase. Moreover, the DNA fragmentation is one of the typical characteristics of cell death by apoptosis, and may be considered in further investigations.

**Figure 4:**
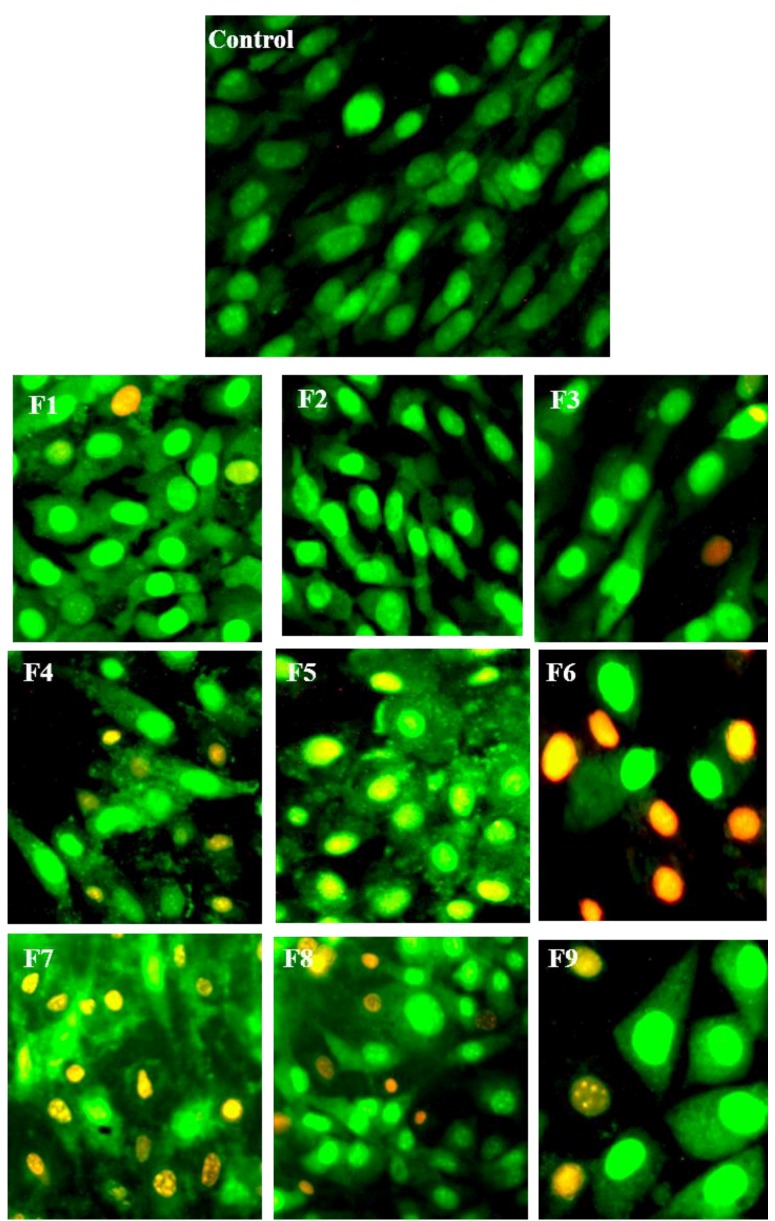
Representative fluorescence microscopic images of cells double-stained with acridine orange (0.3 mg·mL^−1^) and ethidium bromide (1 mg·mL^−1^) after 24 h of exposure to F1–F9. Images of the fields were acquired with a magnification of 200×.

### 3.5. Morphological Identification of Cell Death

To further characterize the cytotoxic effect of SLN formulations, cells were incubated with SLN at CC_50_ concentration and analyzed qualitatively by the AO/EB double-staining assay. This assay is based on the assumption that changes in membrane integrity result in alterations of the cellular permeability to AO and EB dyes. Acridine orange is non-specific and stains in green the nuclei of cells with intact or destabilized membranes. Conversely, ethidium bromide specifically stains in red/orange the nuclei of cells that have a destabilized membrane. Thus, it is possible to identify viable and dead cells (by apoptosis or necrosis) based on the morphology.

**Figure 5:**
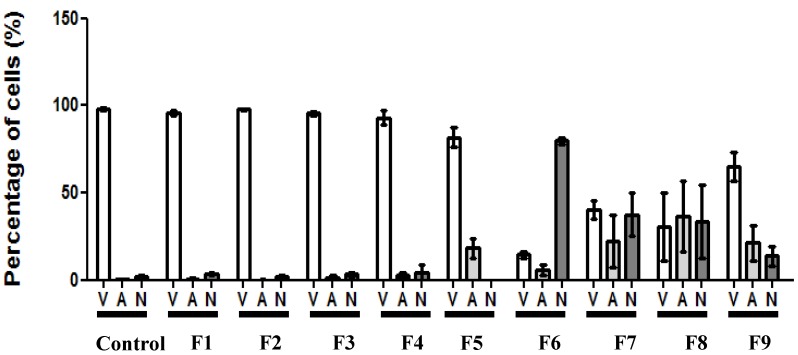
Quantification of viable and dead cells according to morphological identification obtained by double-staining with acridine orange (0.3 mg·mL^−1^) and ethidium bromide (1 mg·mL^−1^) after 24 h of exposition to F1–F9. (V) Viable cells; (A) apoptotic cells; (N) necrotic cells. Data is presented as mean ± SEM from six fields.

Figure 4 shows representative images of fibroblasts treated with each formulation. It was possible to observe the morphology of DNA fragmentation in some of the cells, which is well correlated to the findings in cell cycle analysis (Figure 3). As shown in Figure 5, there was no statistical difference for the formulations F1 to F5 compared to the control (non-treated cells), presenting a similar pattern with a high number of viable cells. On the other hand, formulation F6 presented an increased number of necrotic cells. The cell incubation with SLNs F7 and F8 resulted in reduction of cell viability to a similar value of increased apoptotic and necrotic cells. Although there was also a decrease in viable cells treated with F9, this reduction was not as great as in cells treated with F7 and F8.

### 3.6. Erythrocyte Hemolysis

SLN formulations are attracting increasing attention as colloidal drugs carriers for intravenous application [1]. The hemolytic activity has been suggested as an *in vitro* screening for the toxicity, and also serves as a simple and reliable measurement for estimating the membrane damage caused by formulation *in vivo* [36].

**Figure 6:**
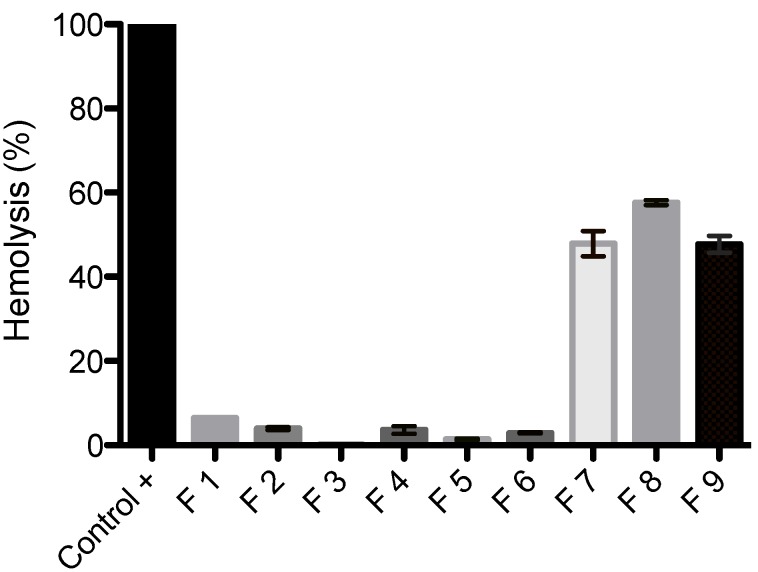
Hemolytic activity of SLNs F1–F9 against human erythrocytes. Blood cells were exposed to SLNs for one hour.

As shown in Figure 6, even after one hour of cell exposure to the SLNs, F1–F6 did not induce hemolytic effects, indicating no detectable disturbance of the red blood cell membranes. On the contrary, F7–F9 showed high hemolytic rate indicating toxicity to blood cell. The F7–F9 formulations resulted in 47.8%, 57.6% and 47.7% of erythrocyte hemolysis, respectively. Overall, a low hemolytic potential of SLNs F1–F6 against erythrocytes was confirmed, suggesting that the type of lipid and/or the shape of the nanoparticles can reduce the interaction of blood components. In addition, it is worth to noting that the tested samples were exposed to erythrocytes for one hour and this is unlikely to happen *in vivo*.

## 4. Conclusions

In this work, a set of nine different drug-free SLN formulations comprising three different matrix lipids and surfactants in varying concentrations was prepared and characterized. The SLNs prepared with stearic acid as the matrix lipid seem to have a pearlized appearance, which impaired the DLS measurements by diffusing the light. On the other hand, DLS measurements were reproducible when tripalmitin or glycerol monostearate were used as the main lipid: the size of SLNs was reduced when surfactant concentration increased. The SLNs F1–F6 showed long-term stability, suggesting good shelf-life behavior of these pre-formulations. At similar concentrations of lipid and surfactant, F7–F9 (stearic acid SLNs) showed higher cytotoxicity in fibroblasts, promoting cell death when compared to F1–F6 (produced with tripalmitin or glycerol monostearate). This finding may be related to lipid type and shape of the nanoparticles. Indeed, the death caused by SLNs F7–F9 was characterized as apoptosis (after 24 hour-exposure, 500 µg lipid·mL^−1^), confirmed by fluorescent images and hemolysis of erythrocytes. However, none of the formulations changed the cell cycle, and there was an increased presence of subdiploid DNA content leading to DNA fragmentation. The mechanism of the cytotoxicity of squared-rod shape nanoparticles needs further investigation. Altogether, the results indicate higher toxicity of SLNs prepared with stearic acid and strongly suggest that the components of the formulation should be analyzed in combination rather than separately to avoid any misinterpretation of the results.
